# Sarcopenia and Myosteatosis Assessed by Magnetic Resonance Enterography May Predict Negative Outcomes in Patients with Crohn’s Disease

**DOI:** 10.5152/tjg.2023.22644

**Published:** 2023-08-01

**Authors:** Rasim Eren Cankurtaran, Yasin Celal Güneş, Emre Dirican, Oktay Algın, Damla Cankurtaran, Öykü Tayfur Yürekli

**Affiliations:** 1 Department of Gastroenterology, Ankara Yıldırım Beyazıt University Faculty of Medicine, Ankara, Turkey; 2 Department of Radiology, Ankara Ataturk Sanatorium Training and Research Hospital, Ankara, Turkey; 3 Department of Medical Informatics and Biostatistics, Mustafa Kemal University Faculty of Medicine, Hatay, Turkey; 4 Department of Radiology, Ankara University Faculty of Medicine, Ankara, Turkey; 5 Department of Physical Medicine and Rehabilitation, University of Health of Sciences Dışkapı Education and Research Hospital, Ankara, Turkey

**Keywords:** Crohn’s disease, sarcopenia, myosteatosis, magnetic resonance enterography, negative outcomes

## Abstract

**Background/Aims:**

Limited research has examined the clinical consequences of sarcopenia and myosteatosis in Crohn’s disease. This study aimed to determine the prevalence, risk factors, and effects of sarcopenia and myosteatosis on prognostic outcomes in Crohn’s disease patients who underwent magnetic resonance enterography.

**Materials and Methods:**

This retrospective observational study included 116 Crohn’s disease patients who underwent magnetic resonance enterography between January 2015 and August 2021. Skeletal muscle index was the ratio of the cross-sectional area of skeletal muscles at the L3 vertebral level to the square of the neck in cross-sectional imaging. Sarcopenia was defined as skeletal muscle index <38.5 cm^2^/m^2^ in women and <52.4 cm^2^/m^2^ in men. Myosteatosis was considered positive if the ratio of the mean signal intensity of the psoas muscle to the mean signal intensity of the cerebrospinal fluid was above 0.107.

**Results:**

Among the negative results in the post-procedure follow-up of the patients, a significant increase was observed in the sarcopenia group regarding abscess and the need for surgery (*P* < .05). Anti-tumor necrosis factor initiation was found to be significantly higher in the follow-up than in patients without myosteatosis (*P* = .029). In the multivariate model established with these variables, the presence of sarcopenia in the surgical follow-up was odds ratio = 5.34 (CI: 1.02-28.03, *P* = .047) and was found to be significantly associated with the increased risk.

**Conclusions:**

The presence of myosteatosis and sarcopenia detected in magnetic resonance enterography may be a harbinger of negative outcomes in Crohn’s disease patients. Nutritional support should be provided to these patients with the potential to alter the course of the disease.

**Main Points**

- Sarcopenia is defined as a decrease in muscle strength, quantity and quality of muscle, and physical performance.
- Sarcopenia is associated with adverse outcomes such as hospitalization, abscess, and surgery in the follow-up of Crohn’s disease (CD) patients.
- Myosteatosis is defined as inter or intramyocellular muscle lubrication that can be detected by imaging methods.
- The presence of myosteatosis and sarcopenia may be a harbinger of negative outcomes in CD patients.
- Nutritional status should also be evaluated in the diagnosis and follow-up of CD patients, and the awareness of clinicians on this issue should be increased.

## Introduction

Crohn’s disease (CD) is a chronic, immune-related, progressive gastrointestinal disease, the causes of which remain unknown. The incidence and prevalence of the disease have increased in Western countries in recent years.^1^ During the course of the disease, the need for hospitalization and surgical intervention may be observed with significant limitations in the quality of life. Patients with CD have been shown to have a 50% higher mortality risk than the normal population.^2^ Progressive bowel damage is thought to be associated with complications in CD patients. Intense efforts have been put into developing noninvasive parameters to predict the extent of bowel damage in clinical follow-up with these patients.

Sarcopenia is defined as a decrease in muscle strength, quantity and quality of muscle, and physical performance.^3^ Recent studies have suggested that the presence of sarcopenia is associated with adverse outcomes such as hospitalization, abscess, and surgery in the follow-up of CD patients.^4^ Studies that investigated the postoperative complications in CD patients reported that the presence of sarcopenia and low psoas muscle area were significantly associated with postoperative complications.^5,6^

Although many methods such as questionnaires, cross-sectional imaging methods, dual-energy x-ray absorptiometry, and physical tests are used in clinical practice to detect the presence of sarcopenia, magnetic resonance imaging (MRI) and computed tomography (CT) are the gold standard non-invasive methods for demonstrating muscle mass and amount.^7^ Both cross-sectional imaging methods are used in the diagnosis and follow-up of CD patients. The MRI is considered a more advantageous cross-sectional method compared to CT, as it gives multi-slice images, has high contrast resolution, and does not contain ionizing radiation.^8^ The European Crohn’s and Colitis Organization guideline recommends cross-sectional imaging methods as a complement to endoscopy in detecting the behavior and spread of the disease. Among these methods, magnetic resonance enterography (MRE) has been primarily recommended due to it’s radiation free nature.^9^

Myosteatosis is defined as inter or intramyocellular muscle lubrication that can be detected by imaging methods.^10^ Although the relationship of myosteatosis with many diseases has been investigated in recent years,^11,12^ limited research has examined the clinical consequences of myosteatosis in CD.^6^

Therefore, this study aimed to determine the prevalence, risk factors, and effects of sarcopenia and myosteatosis on prognostic outcomes in CD patients who underwent MRE after the diagnosis or during the follow-up.

## Materials and Methods

### Demographics of the Patients and Inclusion/Exclusion Criteria

This retrospective observational study included 116 adult CD patients, who were diagnosed based on the suitable workup in the gastroenterology clinic of a single tertiary center and who underwent MRE between January 2015 and August 2021 both for diagnostic and follow-up purposes. The diagnosis of CD was made according to clinical, endoscopic, and histological data. Patients whose demographic data and laboratory values could be accessed within 2 weeks of MRE imaging results were included in the study. However, patients with severe organ failure or sepsis, a history of steroid therapy due to non-inflammatory bowel diseases, and/or patients who were lost to follow-up after imaging were excluded from the study.

### Data Collection

Data about patients’ demographic information, height, weight, body mass index (BMI), smoking habit, surgical history (intestinal or anal), medications, disease characterization according to Montreal classification,^13^ Crohn’s Disease Activity Index (CDAI), and Harvey-Harvey Index (HBI) in line with the severity of the disease and laboratory data [C-reactive protein (CRP), erythrocyte sedimentation rate (ESR), albumin, hemoglobin (Hb), vitamin D] were extracted both from hospital’s electronic data and personal physician files at the time of MRE procedure. At that time, researcher was blind to MRE results. Furthermore, the need for hospitalization, abscess development, need for surgery, need for steroids, the first prescription of biological agents such as anti-tumor necrosis factor (TNF), and switching or dose optimization of anti-TNF agents in the post-MRE follow-up of the patients were recorded as prognostic results.

### Evaluation of Magnetic Resonance Enterography Data

All MRE images were evaluated by experienced radiologists (15-year experienced O.A. and 4-year experienced Y.C.G.) together. Although the radiologists were not informed about the demographic information and laboratory results of the patients, they were informed about the purpose of the study.

Total psoas area (TPA), skeletal muscle area (SMA), cerebrospinal fluid (CSF) intensity, spleen (S) intensity measurements, and the ratios of these values were obtained on axial T2-weighted (T2-SSFSE) and high-resolution T1-weighted 3-dimensional gradient-echo (liver acquisition with volume acceleration [LAVA]) with 2-point Dixon fat/water separation data for the evaluation of sarcopenia. These evaluations were made using the picture archiving and communication system (PACS) system of our hospital and the dedicated workstation of the MR unit (AW Volumeshare 7, GE, USA). Patients with excessive artifacts and narrow spinal canals were excluded from the study.

**Figure 1.**
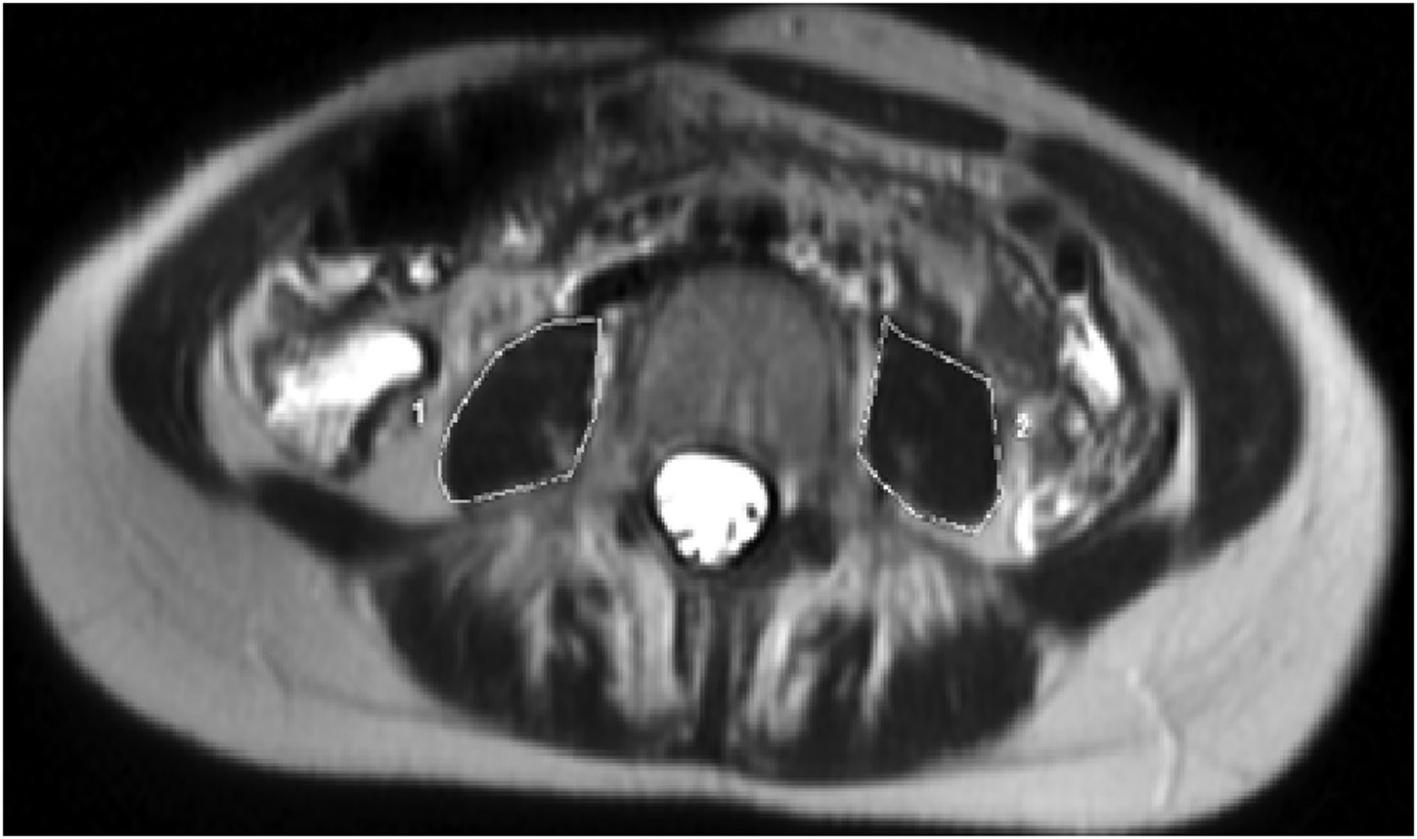
Demonstration of total psoas area calculation on an axial T2-weighted image.

**Total Psoas Area:** It was calculated to pass through the outer contours of the muscle planes with the free-hand region of interest (ROI) method on the axial sections passing through the L3 vertebra level. In the section passing through the same level, both psoas measurements were made separately, and the TPA was measured by summing these values (Figure 1).

**Figure 2.**
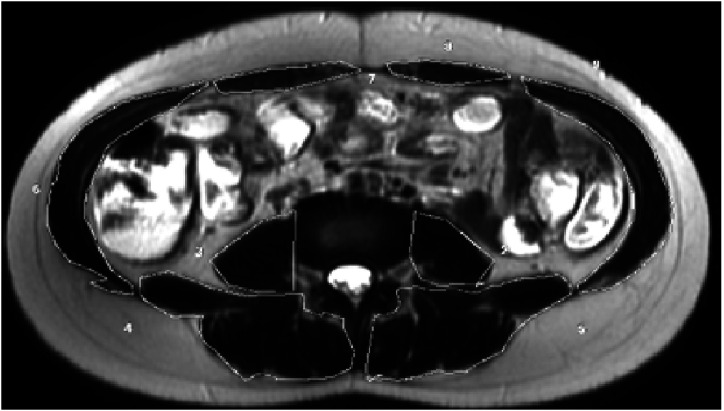
Skeletal muscle area was calculated at the same level with psoas muscle at L4 vertebrae on the axial T2-weighted images.

**Skeletal Muscle Area:** These measurements were made to include all abdominal muscles with the free-hand ROI method on the same slice with TPA measurement (Figure 2).

**Figure 3.**
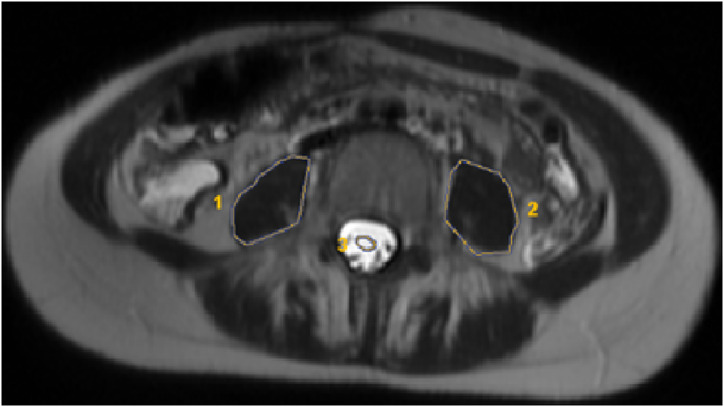
The CSF and psoas intensity were measured with the free-hand ROI method at the same level on the axial T2-weighted images. CSF, cerebrospinal fluid; ROI, region of interest.

**Cerebrospinal Fluid Intensity Measurement: **It was made with the free-hand ROI method on the same slice with TPA measurement, excluding the cauda equina nerve roots (Figure 3).

**Figure 4.**
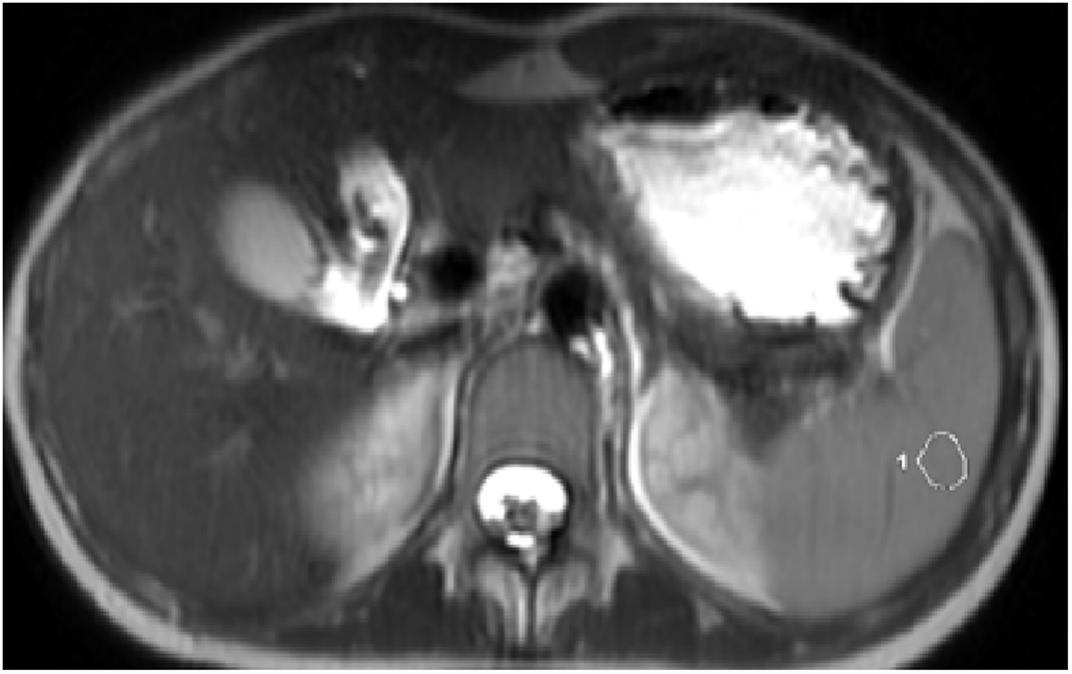
Demonstration of spleen intensity calculation on the axial T2-weighted image.

**Spleen Intensity Measurement:** It was measured by the free-hand ROI method in the parenchyma of the spleen excluding main vessels on the axial image passing through the lower pole of the spleen (Figure 4).

### Definition of Sarcopenia and Myosteatosis

Skeletal muscle index (SMI) (cm^2^/m^2^) was used to define sarcopenia as the ratio of cross-sectional area of skeletal muscles at the L3 vertebral level to the square of the neck in cross-sectional imaging. Sarcopenia was defined as SMI <38.5 cm^2^/m^2^ in women and <52.4 cm^2^/m^2^ in men as suggested by a previous study.^14^

Evaluation of myosteatosis on MRE images is more difficult and complex. Magnetic resonance spectroscopy or chemical shift imaging is required to accurately assess myosteatosis on MR images. In our study, the ratio of the mean signal intensity of the psoas muscle to the mean signal intensity of the CSF was calculated and used to evaluate myosteatosis. Myosteatosis was considered positive if this rate was above 0.107.^6^ In addition, we used the ratio of the mean signal intensity of the psoas muscle to the mean signal intensity of the spleen to evaluate myosteatosis.

### Statistical Analysis

Statistical Package for the Social Sciences version 21.0 (IBM Corp., Armonk, NY, USA) and “corrplot v0.92” package, and R Studio (4.0.5) programs were used for data analysis. Descriptive statistics were presented as mean, standard deviation, median, minimum, maximum, frequency, and percentage values. Shapiro–Wilk and Kolmogorov–Smirnov tests were used to check whether the data were normally distributed. Student’s *t*-test and Mann–Whitney U-tests were used to compare numerical variables by groups. Chi-square (Pearson, Yates, Fisher’s exact, Likelihood ratio) tests were used to analyze the relationships between categorical variables. Pearson and Spearman correlation coefficients and their significance were included to assess the relationships between continuous variables. As for the factors affecting surgery during follow-up, first univariate predictors and then stepwise multivariate logistic regression analysis was performed. Odds ratio (OR) and 95% confidence intervals (CI) were calculated for all variables included in the model. A *P*-value of .05 for all tests was considered statistically significant.

### Ethical Statement

Ethical approval was received from the Ethics Committee of Ankara City Hospital (approval number: E2-21-69273) and the study was conducted in accordance with the Declaration of Helsinki guidelines. Signed informed consent was obtained from each participant prior to the study.

## Results

A total of 116 patients, 57 of whom were (49.1%) women and 59 were (50.9%) men, with a mean age of 37.2 ± 12.2 years, were included in our study. Table 1 shows the baseline characteristics of these patients during the MRE procedure and during follow-up.

The groups according to whether they had sarcopenia or myosteatosis were homogeneous in terms of gender, age, disease duration, and smoking status (*P* > .05). No significant differences were found between the groups in disease activity according to the HBI scores. The CDAI scores of the sarcopenia group were significantly higher than those of the non-sarcopenia group (*P* = .002) (Table 2).

Regarding the variables of the Montreal classification, no significant differences were found between the variables according to the presence or absence of sarcopenia (*P* > .05). In terms of disease location and disease, behavior was significantly different between the myosteatosis and non-myosteatosis groups (*P* = .010, *P* = .003, respectively).

While the laboratory data did not differ significantly regarding the sarcopenia status except for CRP (*P* = .048), CRP and ESR were significantly higher (*P* = .003, *P* = .025, respectively) and albumin was significantly lower (*P* = .017) in the myosteatosis group compared to the group without myosteatosis. On the contrary, Hb and vitamin D levels were not found to be statistically significant between the groups in terms of myosteatosis (*P* > .05).

**Figure 5.**
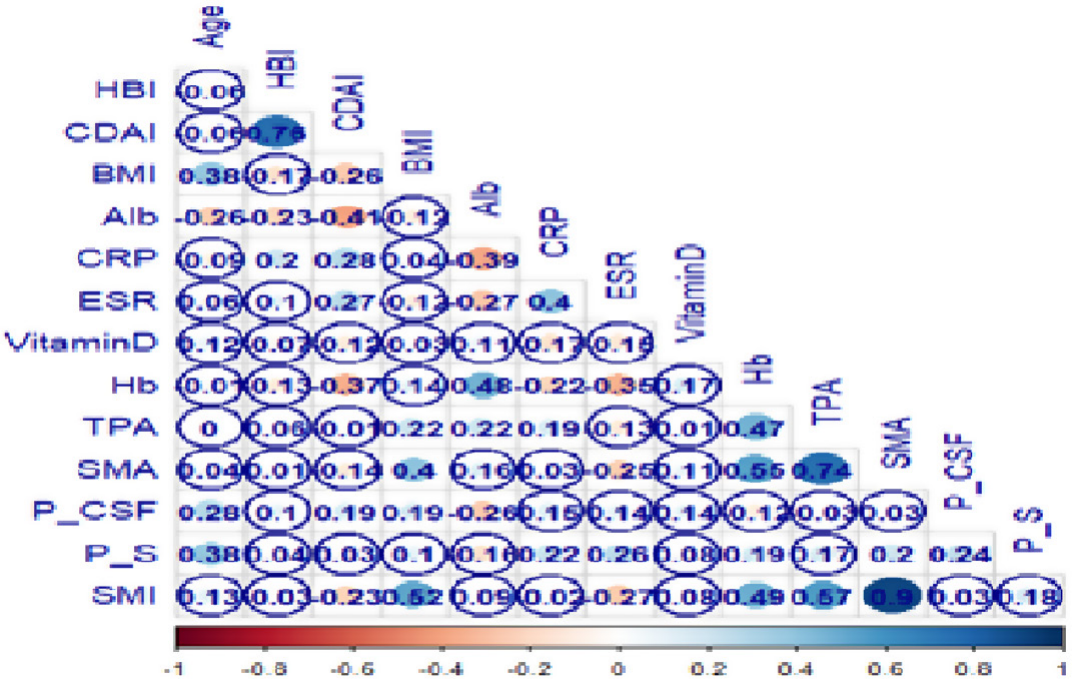
Correlogram of clinical, laboratory, and body composition parameters of patients.

While BMI was found to be significantly lower in patients with sarcopenia, it was found to be significantly higher in patients with myosteatosis (*P* < .001, *P* = .025, respectively). The drugs used during the procedure were homogeneously distributed among the groups (*P* > .05). Among the negative results in the post-procedure follow-up of the patients, a significant increase was observed in the sarcopenia group regarding abscess and the need for surgery (*P* = .045, *P* = .022, respectively) (Table 2). In terms of myosteatosis, anti-TNF initiation was found to be significantly higher in the follow-up than in patients without myosteatosis (*P* = .029) (Table 2). As shown in the correlogram (Figure 5), SMI values and BMI (*r* = 0.52, *P* < .001), SMA (*r* = 0.90, *P* < .001), TPA (*r* = 0.57, *P* < .001), and Hb (*r* = 0.51, *P* < .001) were positively correlated while a negative correlation was found with CDAI (*r* = −0.23, *P* < .012). A positive correlation between P/CSF values and age (*r* = 0.28, *P* < .001), CDAI (*r* = 0.19, *P* < .041), and BMI (*r* = 0.19, *P* < .040) was found and a negative correlation was found between P/CSF values and Alb (*r* = −0.20), *P* < .004). A positive correlation was found between P/S ratio with age (*r* = 0.38, *P* < .001) and CRP (*r* = 0.22, *P* = .04), ESR (*r* = 0.26, *P* = .03), Hb (*r* = 0.19, *P* = .046), SMA (*r* = 0.2, *P* = .041), and P/CSF (*r* = 0.24, *P* = .035). Furthermore, a positive correlation was found between SMI values and BMI (*r* = 0.52, *P* < .001), TPA (*r* = 0.57, *P* < .001), and Hb (*r* = 0.51, *P* < .001) and a negative correlation with ESR (*r* = −0.26, *P* < .004) (Figure 5).

According to the results of the univariate logistic regression model of the variables that were considered to affect the rate of surgery during the follow-up, CDAI, HBI scores, disease behavior, and sarcopenia were found to be significant. Following that, in the multivariate model established with these variables, the presence of sarcopenia in the surgical follow-up was OR = 5.34 (CI: 1.02-28.03, *P* = .047) and the disease behavior particularly the B2 category variable OR = 8.89 (CI: 1.69-46.75, *P* = .010) were found to be significantly associated with the increased risk (Table 3).

## Discussion

This study revealed that sarcopenia and myosteatosis can be detected by MRE, an imaging method routinely used in the diagnosis and follow-up of CD patients. Furthermore, this study concluded that SMA and TPA ratios can be measured to detect sarcopenia, and P/CSF and P/S intensity ratios can be measured to detect myosteatosis using MRE. In the follow-up, some negative prognostic outcomes were found to be higher in patients with sarcopenia and myosteatosis than in other patients.

For many years, sarcopenia was believed to be mainly associated with age and was considered a disease of the elderly population.^15^ However, in recent years, consensus on sarcopenia has divided sarcopenia into 2 types: primary and secondary. Sarcopenia related to aging was defined as primary, whereas sarcopenia due to chronic diseases, inflammation, malignancy, and organ failure was defined as secondary.^16^ It is known that CD and sarcopenia frequently coexist. In recent studies, the prevalence of sarcopenia in CD patients was found to be between 38% and 58%.^4,6,17,18^ Similar rates were found in our study, and sarcopenia was found in 53.5% of our patients.

In a study that investigated sarcopenia in CD patients, no correlation was found between age and sarcopenia.^17^ Similarly, no significant relationship was found between sarcopenia and age in our study. This can be due to the young age of our study population and the cause of sarcopenia may stem from a secondary cause other than age. In addition, no relationship was found between sarcopenia and gender, smoking, and disease duration in our study. Many studies have been conducted on the effects of gender on sarcopenia. One study found the rate of sarcopenia significantly higher in women with CD.^17^ However, Lee et al^18^ suggested that sarcopenia was significantly more common in men. Another study reported no significant relationship between genders in line with this study.^4^ These differences may be due to the fact that SMI used to define sarcopenia is based on a different cut-off value in each study and the cut-off values determined for genders vary in different studies. Previous studies used both scoring indices to examine the relationship between disease activity and sarcopenia in patients with CD. In a study that classified disease severity according to CDAI, no correlation was found between disease severity and sarcopenia.^17^ No significant relationship was found between disease activity and sarcopenia based on HBI.^4^ Our study found no significant differences in patients with sarcopenia according to HBI. However, we found significantly higher CDAI scores in patients with sarcopenia. Furthermore, this study revealed a significant negative relationship between CDAI and SMI with the correlation test. No relationships between the disease behavior and localization and sarcopenia were found. These results overlap with the findings reported by the 2 studies mentioned earlier.^4,17^

Previous research focused on the relationship between laboratory results and sarcopenia in patients with CD.^4,18,19^ Two studies reported a significant relationship between albumin levels and sarcopenia.^4,19^ In this study, however, none of the laboratory values except for CRP were found to be significantly associated with sarcopenia. These findings are similar to those of Lee et al.^18^ Higher CRP levels in the sarcopenia group suggest that sarcopenia may be more severe in patients with more active disease. However, the correlation between the sarcopenia marker SMI and CRP was not significant.

In this study, the mean BMI of sarcopenic patients was within the normal range, but the mean BMI of sarcopenic patients was found to be significantly lower than those without sarcopenia. In line with this study, previous studies found the BMI of patients with sarcopenia to be significantly lower.^4,17^ On the other hand, several studies indicated that most sarcopenic patients have normal BMI, and some may even be obese.^3,20,21^ These discrepancies may be because BMI is an indicator that reflects not only muscle mass but also body fat percentage.

Previous studies have often focused on the negative effects of sarcopenia on postoperative outcomes.^6,22^ Conflicting results were reported by 2 studies evaluating whether the presence of sarcopenia detected by CT causes negative prognostic results. Grillot et al^4^ found significantly higher rates of hospitalization, abscess, and surgery in the follow-up of patients with sarcopenia. Lee et al.^18^ on the other hand, suggested that the presence of sarcopenia has no effect on prognostic outcomes. In a review study derived from 5 studies, most of which consisted of CD patients, it was stated that the presence of sarcopenia may predict the need for surgical intervention.^23^ In this study, sarcopenia was found to be significantly higher in patients with surgery and abscess during follow-up.

The other pathology that our study investigated was the presence of myosteatosis and its effects on prognosis. Few studies have been conducted on CD regarding the effect of myosteatosis on postoperative complications.^6,24^ In a study including 348 operated CD patients, advanced age, female gender, and increased BMI were found to be significantly higher in the patients with myosteatosis.^24^ It was found that BMI was significantly higher in patients with myosteatosis compared to patients without myosteatosis in our study. A comprehensive study evaluating myosteatosis with CT reported proinflammatory cytokines and CRP to be significantly higher in patients with myosteatosis.^25^ Significantly higher CRP and ESR in myosteatosis patients were found in our study, which was consistent with the findings of this study.^25^ In the correlation test, our study revealed that CRP and ESR were significantly positively correlated with the P/S ratio, not the P/CSF ratio. Two studies evaluating the effect of myosteatosis on postoperative outcomes in CD patients also suggested that myosteatosis does not have a significant prognostic value. In line with our study, these 2 studies evaluated the fat intensity ratio with MRE.^6,24^ In our study, initiation of only anti-TNF was found to be significantly higher in patients with myosteatosis. Based on current literature data, it seems difficult to predict the reason for the higher incidence of anti-TNF initiation, which is an unfavorable prognostic outcome, in patients with myosteatosis, the higher secretion of the proinflammatory cytokines mentioned earlier in myosteatosis patients may have resulted in a high inflammatory burden necessitating the use of more potent anti-inflammatory therapies.

The findings of this study suggest the presence of sarcopenia, disease activity, and disease behavior were among the most important risk factors affecting the rate of surgery. Grillot et al^4^ also stated that the presence of sarcopenia is an independent risk factor for the surgery. These findings indicate that early diagnosis of sarcopenia and subsequent nutritional support are very important for follow-up of the patients and may have the potential to alter the course of the disease.

One of the strengths of our study is that it sheds light on the effect of sarcopenia and myosteatosis on the prognostic outcomes of CD patients given the limited literature on the prevalence and prognostic results of myosteatosis in CD. Furthermore, this study showed that sarcopenia and myosteatosis can be detected by experienced radiologists with MRE, which is an imaging method frequently used in the diagnosis and follow-up of CD patients. This detection method can serve as an important noninvasive marker to predict the course of the method.

This study had 2 main limitations. First, this study employed a retrospective design in a single center. However, our center is a tertiary university hospital where more than 800 patients with inflammatory bowel diseases are followed, approximately 350 of whom are CD patients. Medical and surgical treatment decisions of the patients are made in accordance with international guidelines and by a multidisciplinary council decision. Due to being a referral center patient with a more aggressive disease may have been included in this study. Second, there may be variations due to ethnic differences between the SMI cut-off values of the patients in the study, which we refer to define sarcopenia, and the patient population.

In conclusion, the presence of myosteatosis and sarcopenia detected in MRE may be a harbinger of negative outcomes in CD patients. Nutritional status should also be evaluated in the diagnosis and follow-up of CD patients, and the awareness of clinicians on this issue should be increased. If necessary, nutritional support should be provided to these patients with the potential to alter the course of the disease.

## Figures and Tables

**Table 1.** Baseline Characteristics

Baseline Characteristics During Imaging	(n = 116)
Demographic and clinical data	
Male	59 (50.9)
Age (years)	38.6 ± 12.9
Current smokers	42 (36.2)
Disease duration (months)	32 (15-312)
CDAI	153.5 (8-438)
HBI	5 (2-14)
Montreal classification^*^	
Age at diagnosis	
A1	5 (4.3)
A2	77 (66.4)
A3	34 (29.3)
Location	
L1	81 (69.8)
L2	7 (6.1)
L3	28 (24.1)
Behavior	
B1	76 (66.5)
B2	21 (18.1)
B3	19 (16.4)
Perianal disease	10 (8.6)
Laboratory data	
Hemoglobin (mg/dL)	13.5 ± 1.8
Albumin (mg/dL)	4.5 (2.8-5.4)
ESR (mm/h)	8 (3-56)
CRP (mg/dL)	3.95 (0.4-193.0)
Vitamin D (ng/mL)	16.2 ± 6.9
Body composition and radiological data	
BMI (kg/m^2^)	23.9 ± 3.8
SMI (cm^2^/m^2^)	45.6 ± 9.9
TPA (cm^2^)	21.5 ± 8.8
SMA (cm^2^)	128.7 ± 33.1
P/CSF ratio	0.09 ± 0.02
P/S ratio	0.39 (0.20-0.90)
Treatments during MR procedure (some patients were taking more than 1 medication)	
Budesonide	35 (30.2)
Oral/IV corticosteroids	9 (7.8)
Antibiotics	10 (8.6)
Thiopurine	28 (24.1)
Oral 5-ASA	31 (26.7)
Anti-TNF	32 (27.6)
Surgery (anal + intestinal)	20 (17.2)
Follow-up	
Follow-up (months)	12 (12-79)
Hospitalization	29 (25.0)
Abscess	8 (6.9)
Surgery (perianal + intestinal)	14 (12.1)
Start of an anti-TNF	35 (30.2)
Switch off anti-TNF	7 (6.0)
Optimization/intensification of anti-TNF	15 (12.9)
Start of anti-adhesion molecules	8 (6.9)

ASA, aminosalicylates; BMI, body mass index; CDAI, Crohn’s Disease Activity Index; CRP, C-reactive protein; CSF, cerebrospinal fluid; ESR, erythrocyte sedimentation rate; HBI, Harvey Bradshaw Index; P, psoas; S, spleen; SMA, skeletal muscle area; SMI, skeletal muscle index; TNF, tumor necrosis factor; TPA, total psoas area.

Numeric variables with normally distribution, continuous were expressed as mean ± standard deviation, other continuous variables with median (min-max) and categorical variables with n (%).

*According to the Montreal classification: A1, <17; A2, 17-40; A3, >40; L1, ileal; L2, colonic; L3, ileocolonic; B1, non-stricturing non-penetrating; B2, stricturing; B3, penetrating.

**Table 2.** Comparison of No-Sarcopenia and Sarcopenia and No-Myosteatosis and Myosteatosis Patient Groups in Terms of Their Demographics, Clinical, Laboratory, Radiological Characteristics, and Adverse Outcomes at Follow-Up

Variables		No-Sarcopenia (n = 54)	Sarcopenia (n = 62)	*P*	No-Myosteatosis (n = 81)	Myosteatosis (n = 35)	*P*
Gender (male)		25 (46.3)	34 (54.8)	.359	46 (56.8)	13 (37.1)	.08
Age		37.7 ± 10.8	36.8 ± 13.3	.684	35.9 ± 12.1	40.1 ± 12.1	.08
Disease duration (months)		34 (15-260)	31 (16-312)	.965	32 (15-186)	40 (18-312)	.239
Smoking (yes)		20 (37)	22 (35.5)	.862	27 (33.3)	15 (42.9)	.442
CDAI		139.1 ± 71.8	186.7 ± 84.3	**.002**	150 (8-329)	154 (33-438)	.261
HBI		5.3 ± 2.5	6.1 ± 2.4	.08	5 (2-11)	5 (2-14)	.956
Montreal classification^*^							
Age at diagnosis				.428			.114
A1		2 (3.7)	3 (4.8)		4 (4.9)	1 (2.9)	
		A2			33 (61.1)	44 (71)		58 (71.6)	19 (54.3)
		A3			19 (35.2)	15 (24.2)		19 (23.5)	15 (42.9)
Location				.383			**.010**
L1		40 (74.1)	41 (66.1)		63 (77.8)	18 (51.4)	
		L2			4 (7.4)	3 (4.8)		5 (6.2)	2 (5.7)
		L3			10 (18.5)	18 (29.0)		13 (16)	15 (42.9)
Behavior				.353			**.003**
B1		38 (70.4)	38 (61.3)		61 (75.3)	15 (42.9)	
		B2			10 (18.5)	11 (17.7)		10 (12.3)	11 (31.4)
		B3			6 (11.1)	13 (21)		10 (12.3)	9 (25.7)
Perianal disease (yes)		4 (7.4)	6 (9.7)	.664	6 (7.4)	4 (11.4)	.486
Laboratory data							
CRP (mg/dL)		3.10 (0.4-113.0)	4.7 (0.5-193.0)	**.048**	3.1 (0.4-193)	5.9 (0.5-113)	**.003**
ESR (mm/h)		8 (3-45)	8 (3-56)	.326	8 (3-56)	16 (3-49)	**.025**
Hemoglobin (mg/dL)		13.7 ± 1.5	13.4 ± 1.9	.183	13.7 ± 1.7	13.1 ± 1.8	.07
Albumin (mg/dL)		4.4 (2.8-5.2)	4.6 (3.3-5.4)	.712	4.5 ± 0.38	4.3 ± 0.5	**.017**
Vitamin D (ng/dL)		14.1 (8-43)	14.2 (4-37)	.890	15.8 ± 6.5	17.3 ± 7.9	.280
Body composition and radiological data							
BMI (kg/m^2^)		25.5 ± 4.1	22.5 ± 2.9	**<.001**	23.4 ± 3.3	25.1 ± 4.6	**.025**
SMI (cm^2^/m^2^)		51.9 ± 9.3	40.2 ± 6.89	**<.001**	45.6 ± 9.5	45.7 ± 11.3	.979
TPA (cm^2^)		21.8 ± 9.1	21.3 ± 8.6	.767	22.4 ± 8.6	22.6 ± 19.4	.092
SMA (cm^2^)		133.5 (75.8-202.2)	119.1 (65.7-226.9)	**<.001**	130.3 ± 32.7	125.3 ± 34.5	.547
P/CSF ratio		0.1 (0.03-0.2)	0.09 (0.07- 0.17)	.766	0.08 ± 0.01	0.12 ± 0.01	**<.001**
P/S ratio		0.4 (0.23-0.79)	0.39 (0.2-0.9)	.503	0.37 (0.2-0.8)	0.41 (0.3-0.9)	**.003**
Treatments during MR procedure							
Budenoside		17 (31.5)	18 (29.0)	.774	26 (32.1)	9 (25.7)	.492
Oral/IV corticosteroids		4 (7.4)	5 (8.1)	1.00	6 (7.4)	3 (8.6)	1.00
Antibiotics		4 (7.5)	6 (9.7)	.749	4 (4.9)	6 (17.1)	.064
Thiopurine		16 (29.6)	12 (19.4)	.197	19 (23.5)	9 (25.7)	.980
Oral 5-ASA		16 (29.6)	15 (24.2)	.653	23 (28.4)	8 (22.9)	.650
Anti-TNF		17 (31.5)	15 (24.2)	.381	20 (24.7)	12 (34.3)	.404
Surgery (anal + intestinal)		8 (14.8)	12 (19.4)	.690	10 (12.3)	10 (28.6)	.058
Adverse outcomes at follow-up							
Follow-up (months),		23.5 (12-79)	24.5 (15-76)	.290	24 (13-79)	24 (12-76)	.509
Hospitalization		12 (22.2)	17 (27.9)	.486	19 (23.8)	10 (28.6)	.753
Abscess		1 (1.9)	7 (11.3)	**.045**	5 (6.2)	3 (8.6)	.696
Surgery (perianal + intestinal)		2 (3.7)	12 (19.4)	**.022**	10 (12.3)	4 (11.4)	1.00
Start of an anti-TNF		13 (24.1)	22 (35.5)	.182	19 (23.5)	16 (45.7)	**.029**
Switch off anti-TNF		2 (3.7)	5 (8.1)	.447	4 (4.9)	3 (8.6)	.429
Optimization/intensification of anti-TNF		7 (13.0)	8 (12.9)	.992	8 (9.9)	7 (20.7)	.145
Start of anti-adhesion molecules		4 (7.4)	4 (6.5)	1.000	3 (3.7)	5 (14.3)	.053

ASA, aminosalicylates; BMI, body mass index; CDAI, Crohn’s Disease Activity Index; CRP, C-reactive protein; CSF, cerebrospinal fluid; ESR, erythrocyte sedimentation rate; HBI, Harvey Bradshaw Index; P, psoas; S, spleen; SMA, skeletal muscle area; SMI, skeletal muscle index; TNF, tumor necrosis factor; TPA, total psoas area.

Numaric variables with normally distribution, continuous were expressed as mean ± standard deviation, other continuous variables with median (min-max) and categorical variables with n (%).

*According to the Montreal classification: A1, <17; A2, 17-40; A3, >40; L1, ileal; L2, colonic; L3, ileocolonic; B1, non-stricturing non-penetrating; B2, stricturing; B3, penetrating.

**Table 3.** Univariate and Multivariate Analysis of Variables Considered to Affect the Rate of Surgery During Follow-Up

Variables		Univariate Analysis			Multivariate Analysis (Adjusted)		
		Odds Ratio	95% CI	*P*	Odds Ratio	95% CI	*P*
Gender (male)		1,33	0.43-4.11	.617			
Age		0.98	0.940-1.03	.487			
CDAI		1.01	1.003-1.017	**.004**	0.99	0.98-1.01	.851
HBI		1.36	1.095-1.693	**.006**	1.27	0.86-1.89	.220
Behavior^*^							
B1							
		B2		9.73	2.18-43.32	**.003**	**8.89**	**1.69-46.75**	**.010**
		B3		8.69	1.86-40.59	**.006**	4.98	0.94-26.25	.058
CRP (mg/dL)		1.00	0.98-1.02	.971			
ESR (mm/h)		0.96	0.904-1.02	.208			
Hemoglobin (mg/dL)		1.16	0.84-1.61	.361			
Albumin (mg/dL)		1.10	0.301-4.02	.885			
Vitamin D (ng/dL)		0.99	0.92-1.08	.962			
BMI (kg/m^2^)		0.95	0.81-1.1	.552			
SMI (cm^2^/m^2^)		0.98	0.92-1.04	.572			
TPA (cm^2^)		0.98	0.92-1.05	.701			
SMA (cm^2^)		1.003	0.98-1.02	.744			
P/S ratio		1.48	0.017-132.3	.862			
Sarcopenia		6.24	1.32-29.29	**.020**	**5.34**	**1.02-28.03**	**.047**
Myosteatosis		0.916	0.26-3.14	.889			

BMI, body mass index; CDAI, Crohn’s Disease Activity Index; CRP, C-reactive protein; CSF, cerebrospinal fluid; ESR, erythrocyte sedimentation rate; HBI, Harvey Bradshaw Index; P, psoas; S, spleen; SMA, skeletal muscle area; SMI, skeletal muscle index; TPA, total psoas area.

^*^According to the Montreal classification: B1, non-stricturing non-penetrating; B2, stricturing; B3, penetrating.
